# 5-(1-Benzyl-1*H*-1,2,3-triazol-4-yl)-2,1,3-benzoxadiazole

**DOI:** 10.1107/S1600536812041827

**Published:** 2012-10-13

**Authors:** Jessie A. Key, Christopher W. Cairo, Robert McDonald

**Affiliations:** a Alberta Glycomics Centre, Department of Chemistry, University of Alberta, Edmonton, AB T6G 2G2, Canada; b X-ray Crystallography Laboratory, Department of Chemistry, University of Alberta, Edmonton, AB T6G 2G2, Canada

## Abstract

In the title compound, C_15_H_11_N_5_O, which was prepared as part of a study to identify fluoro­genic substrates for the Cu-catalysed azide–alkyne cyclo­addition (CuAAC) reaction, the benzoxa­diazole unit and the triazole ring are much more closely coplanar [dihedral angle = 10.92 (7)°] than either is to the benzyl group [dihedral angles = 69.13 (3)° and 78.20 (4)°, respectively]. The crystal structure features two different sets of weak inter­molecular C—H⋯N inter­actions between adjacent benzoxadiazole and triazole rings, forming a chain that propagates in the [-110] direction parallel to the *ab* plane.

## Related literature

For the synthesis of the title compound, see: Key & Cairo (2011 ▶). For computational studies of the absorption and fluorescence of the title compound, see: Brown *et al.* (2012 ▶). For structures with 4-aryl substituted 1-benzyl-1,2,3-triazole rings, see: Key *et al.* (2008) ▶; Li *et al.* (2011 ▶); Raghavendra & Lam (2004 ▶); Sarmiento-Sánchez *et al.* (2011) ▶. For two related benzoxadiazole structures, see: Key, Cairo & Ferguson (2012 ▶); Key, Cairo & McDonald (2012 ▶). For the synthesis of analogous triazole-substituted coumarin structures, see: Key *et al.* (2009 ▶). For information on reactive chromophores, see: Cairo *et al.* (2010 ▶). For recent work on small mol­ecule fluoro­phores, see: Lavis & Raines (2008 ▶).
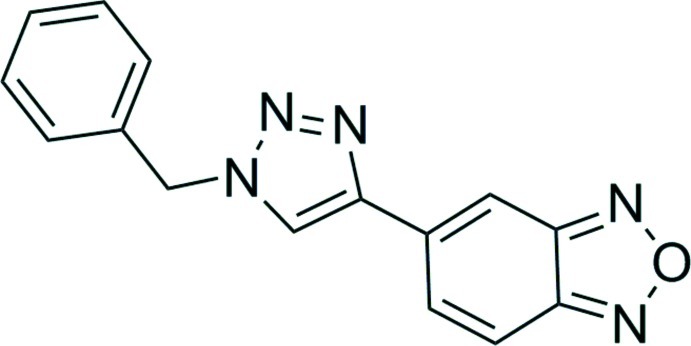


## Experimental

### 

#### Crystal data

C_15_H_11_N_5_O
*M* *_r_* = 277.29
Triclinic, 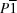

*a* = 5.7526 (4) Å
*b* = 9.9261 (6) Å
*c* = 11.7012 (8) Å
α = 90.3799 (7)°
β = 99.2517 (7)°
γ = 103.2900 (7)°
*V* = 641.14 (7) Å^3^
*Z* = 2
Mo *K*α radiation
μ = 0.10 mm^−1^
*T* = 173 K
0.50 × 0.29 × 0.23 mm

#### Data collection

Bruker APEXII CCD diffractometer
Absorption correction: numerical (*SADABS*; Sheldrick, 2008 ▶) *T*_min_ = 0.953, *T*_max_ = 0.978
5667 measured reflections
2879 independent reflections
2489 reflections with *I* > 2σ(*I*)
*R*_int_ = 0.012

#### Refinement

*R*[*F* ^2^ > 2σ(*F* ^2^)] = 0.035
*wR*(*F* ^2^) = 0.096
*S* = 1.06
2879 reflections
191 parameters
H-atom parameters constrained
Δρ_max_ = 0.23 e Å^−3^
Δρ_min_ = −0.17 e Å^−3^

### 

Data collection: *APEX2* (Bruker, 2008 ▶); cell refinement: *SAINT* (Bruker, 2008 ▶); data reduction: *SAINT*; program(s) used to solve structure: *SHELXD* (Sheldrick, 2008 ▶); program(s) used to refine structure: *SHELXL97* (Sheldrick, 2008 ▶); molecular graphics: *SHELXTL* (Sheldrick, 2008 ▶); software used to prepare material for publication: *publCIF* (Westrip, 2010 ▶).

## Supplementary Material

Crystal structure: contains datablock(s) I, global. DOI: 10.1107/S1600536812041827/mw2083sup1.cif

Structure factors: contains datablock(s) I. DOI: 10.1107/S1600536812041827/mw2083Isup2.hkl

Supplementary material file. DOI: 10.1107/S1600536812041827/mw2083Isup3.cml

Additional supplementary materials:  crystallographic information; 3D view; checkCIF report

## Figures and Tables

**Table 1:** Hydrogen-bond geometry (Å, °)

*D*—H⋯*A*	*D*—H	H⋯*A*	*D*⋯*A*	*D*—H⋯*A*
C5—H5⋯N3^i^	0.95	2.50	3.3483 (15)	148
C8—H8⋯N2^ii^	0.95	2.57	3.4367 (15)	151

Symmetry codes: (i) 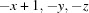; (ii) 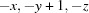.

**Fig. 1.**
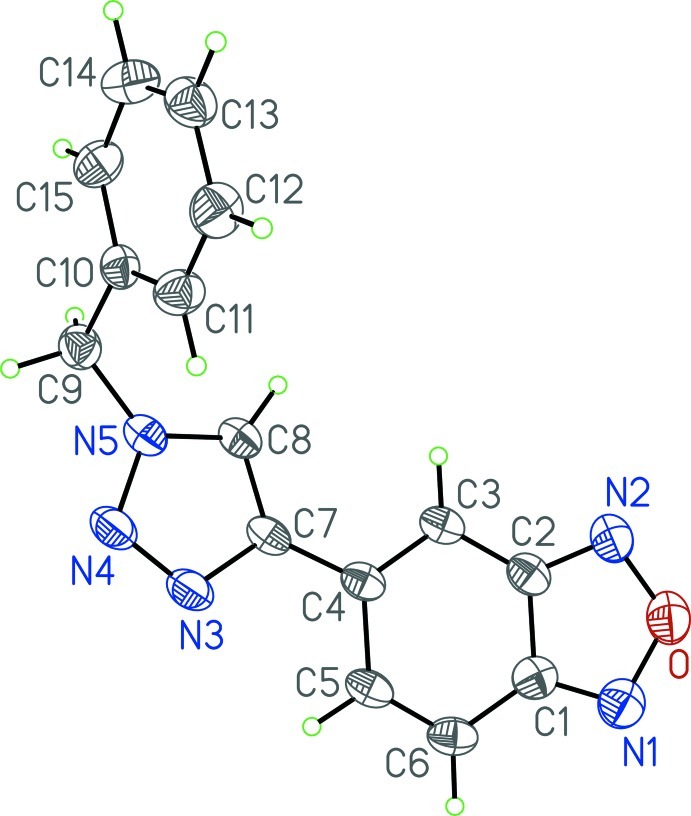
View of I. Non-hydrogen atoms are represented by Gaussian ellipsoids at the 50% probability level. Hydrogen atoms are represented with artificially small thermal parameters.

**Fig. 2.**
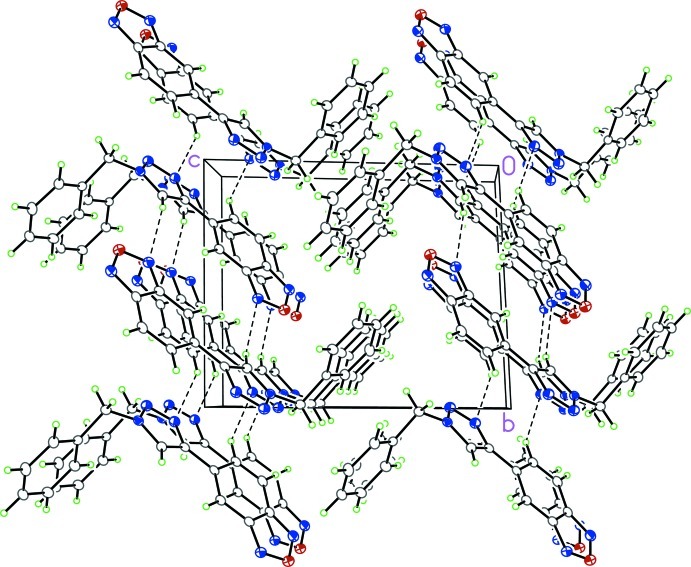
Illustration of crystal packing as viewed parallel to the crystal a axis. Nonbonded C-H···N interactions are shown with dashed lines (see Table 1).

**Fig. 3.**
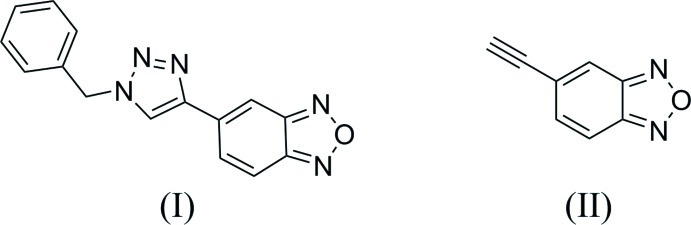
Compounds used in this study.

## supplementary crystallographic information

### Comment

Small molecule fluorophores have significant applications in both chemistry and biology, and new probes are an area of continued research (Lavis & Raines, 2008). Reactive chromophores, or dyes which change their spectral properties upon chemical reaction, have the potential to act as indicators of specific functional groups or enzymatic activity in complex mixtures (Cairo *et al.*, 2010). We examined benzoxadiazole chromophores as substrates for the Cu-catalyzed azide-alkyne cycloaddition (CuAAC) by generating a series of analogs that contained either an alkyne or azide group appended to the ring. The title compound, I, was generated from 4-ethynylbenzoxadiazole (II), and showed a large increase in fluorescence relative to the precursor (Key & Cairo, 2011). As a result, we designated compound II as a fluorogenic substrate for CuAAC.

In the crystal, the dihedral angle between the mean planes of the benzoxadiazole group and the triazole ring is 10.92 (7)°, while the benzyl ring is twisted significantly out of the plane of the other two rings, with dihedral angles of 69.13 (3)° and 78.20 (4)° to the benzoxadiazole and triazole rings, respectively. Two different sets of weak intermolecular C-H···N interactions are observed between adjacent triazole and benzoxadiazole rings related by the inversion centers (^1^/_2_, 0, 0) (2.50 Å for H5···N3[1-*x*, -*y*, -*z*]) and (0, ^1^/_2_, 0) (2.57 Å for H8···N2[-*x*, 1-*y*, -*z*]). A parallel-stacking interaction is observed between benzoxadiazole rings related by the inversion center (^1^/_2_, ^1^/_2_, 0) (interplanar spacing = 3.366 Å).

### Experimental

4-Ethynylbenzoxadiazole (II) (26 mg, 0.18 mmol, 1 equiv) was dissolved in 1:1 water/methanol (5 mL), followed by addition of benzyl azide (0.095 mL, 0.90 mmol, 5 equiv). Copper sulphate (6 mg, 0.036 mmol, 0.2 equiv) and ascorbic acid (10 mg, 0.025 mmol, 0.3 equiv) were then added to the solution. The reaction mixture was allowed to stir at room temperature for 1.5 h turning an opaque white colour. The solvent was removed in vacuo and the crude product was dissolved into chloroform, washed with water, dried over MgSO_4_, and concentrated in vacuo. The compound was purified by column chromatography (EtOAc/hexanes), and obtained as a white powder (30 mg, 60% yield). m.p. 148.7-149.5 °C; ^1^H NMR (400 MHz, CDCl_3_): δ 8.19 (s, 1H), 8.02 (dd, 1H, ^4^*J* = 1.2 Hz, ^3^*J* = 9.6 Hz), 8.01 (dd, 1H, ^4^*J* = 1.2 Hz, ^3^*J* = 9.6 Hz), 7.88 (s, 1H), 7.40-7.48 (m, 3H), 7.34-7.40 (m, 2H), 5.64 (s, 2H); ^13^C NMR (100 MHz, CDCl_3_): δ 149.6, 149.0, 146.2, 133.8, 131.2, 129.6, 129.4, 128.5, 121.4, 117.3, 111.3, 54.8; IR (microscope): ν = 3138, 3122, 3071, 3040, 2924, 2853, 1630, 1564 cm^-1^; ES-HRMS calculated for C_15_H_11_N_5_O [*M*+H]^+^: 278.1036; observed: 278.1032. *R*_f_ = 0.18 (1:3 EtOAc/hexanes).

### Refinement

All H atoms were generated in idealized positions and refined using a riding model with fixed C-H distances (C-H_aromatic_ = 0.95 Å, C-H_methylene_ = 0.99 Å) and with *U*_iso_(H) = 1.2*U*_eq_(C).

### Crystal data

**Table:**

C_15_H_11_N_5_O	*Z* = 2
*M**_r_* = 277.29	*F*(000) = 288
Triclinic, *P*1	*D*_x_ = 1.436 Mg m^−^^3^
Hall symbol: -P 1	Mo *K*α radiation, λ = 0.71073 Å
*a* = 5.7526 (4) Å	Cell parameters from 4623 reflections
*b* = 9.9261 (6) Å	θ = 2.7–27.3°
*c* = 11.7012 (8) Å	µ = 0.10 mm^−^^1^
α = 90.3799 (7)°	*T* = 173 K
β = 99.2517 (7)°	Fragment, colourless
γ = 103.2900 (7)°	0.50 × 0.29 × 0.23 mm
*V* = 641.14 (7) Å^3^	

### Data collection

**Table:**

Bruker APEXII CCD diffractometer	2879 independent reflections
Radiation source: fine-focus sealed tube	2489 reflections with *I* > 2σ(*I*)
Graphite monochromator	*R*_int_ = 0.012
Detector resolution: 8.26 pixels mm^-1^	θ_max_ = 27.3°, θ_min_ = 1.8°
ω scans	*h* = −7→7
Absorption correction: numerical (*SADABS*; Sheldrick, 2008)	*k* = −12→12
*T*_min_ = 0.953, *T*_max_ = 0.978	*l* = −15→15
5667 measured reflections	

### Refinement

**Table:**

Refinement on *F*^2^	Secondary atom site location: difference Fourier map
Least-squares matrix: full	Hydrogen site location: inferred from neighbouring sites
*R*[*F*^2^ > 2σ(*F*^2^)] = 0.035	H-atom parameters constrained
*wR*(*F*^2^) = 0.096	*w* = 1/[σ^2^(*F*_o_^2^) + (0.0444*P*)^2^ + 0.1305*P*] where *P* = (*F*_o_^2^ + 2*F*_c_^2^)/3
*S* = 1.06	(Δ/σ)_max_ < 0.001
2879 reflections	Δρ_max_ = 0.23 e Å^−^^3^
191 parameters	Δρ_min_ = −0.17 e Å^−^^3^
0 restraints	Extinction correction: *SHELXL*, Fc^*^=kFc[1+0.001xFc^2^λ^3^/sin(2θ)]^-1/4^
0 constraints	Extinction coefficient: 0.027 (4)
Primary atom site location: structure-invariant direct methods	

### Special details

**Table:**

Geometry. All standard uncertainties (s.u.'s) (except the s.u. in the dihedral angle between two least-squares planes) are estimated using the full covariance matrix. The cell s.u.'s are taken into account individually in the estimation of s.u.'s in distances, angles and torsion angles; correlations between s.u.'s in cell parameters are only used when they are defined by crystal symmetry. An approximate (isotropic) treatment of cell s.u.'s is used for estimating s.u.'s involving least-squares planes.
Refinement. Refinement of *F*^2^ against ALL reflections. The weighted *R*-factor *wR* and goodness of fit *S* are based on *F*^2^, conventional *R*-factors *R* are based on *F*, with *F* set to zero for negative *F*^2^. The threshold expression of *F*^2^ > σ(*F*^2^) is used only for calculating *R*-factors(gt) *etc*. and is not relevant to the choice of reflections for refinement. *R*-factors based on *F*^2^ are statistically about twice as large as those based on *F*, and *R*- factors based on ALL data will be even larger.

### Fractional atomic coordinates and isotropic or equivalent isotropic displacement parameters (Å^2^)

**Table:**

	*x*	*y*	*z*	*U*_iso_*/*U*_eq_	
O	0.37047 (18)	0.60313 (9)	−0.24598 (8)	0.0488 (3)	
N1	0.5182 (2)	0.51386 (12)	−0.25718 (9)	0.0454 (3)	
N2	0.2225 (2)	0.56215 (11)	−0.16419 (9)	0.0426 (3)	
N3	0.3192 (2)	0.05933 (11)	0.11897 (9)	0.0397 (3)	
N4	0.2143 (2)	−0.00352 (11)	0.20195 (9)	0.0405 (3)	
N5	0.02778 (17)	0.05338 (10)	0.21296 (8)	0.0326 (2)	
C1	0.4623 (2)	0.41788 (12)	−0.18298 (10)	0.0351 (3)	
C2	0.2785 (2)	0.44754 (12)	−0.12510 (10)	0.0337 (3)	
C3	0.1868 (2)	0.36165 (12)	−0.03855 (10)	0.0324 (2)	
H3	0.0650	0.3818	0.0002	0.039*	
C4	0.28041 (19)	0.24890 (11)	−0.01314 (9)	0.0298 (2)	
C5	0.4649 (2)	0.21869 (12)	−0.07346 (10)	0.0340 (3)	
H5	0.5248	0.1388	−0.0539	0.041*	
C6	0.5554 (2)	0.29873 (13)	−0.15607 (10)	0.0370 (3)	
H6	0.6760	0.2766	−0.1946	0.044*	
C7	0.2003 (2)	0.15745 (11)	0.07735 (9)	0.0303 (2)	
C8	0.0138 (2)	0.15328 (12)	0.13786 (10)	0.0332 (3)	
H8	−0.1008	0.2093	0.1285	0.040*	
C9	−0.1176 (2)	0.01211 (12)	0.30362 (10)	0.0354 (3)	
H9A	−0.0691	−0.0676	0.3429	0.042*	
H9B	−0.2903	−0.0184	0.2678	0.042*	
C10	−0.0894 (2)	0.12825 (11)	0.39256 (9)	0.0305 (2)	
C11	0.1235 (2)	0.22895 (13)	0.42168 (11)	0.0382 (3)	
H11	0.2570	0.2280	0.3835	0.046*	
C12	0.1428 (2)	0.33104 (13)	0.50619 (11)	0.0432 (3)	
H12	0.2891	0.4004	0.5251	0.052*	
C13	−0.0481 (2)	0.33292 (13)	0.56299 (10)	0.0416 (3)	
H13	−0.0332	0.4023	0.6218	0.050*	
C14	−0.2610 (2)	0.23349 (15)	0.53388 (11)	0.0442 (3)	
H14	−0.3934	0.2341	0.5729	0.053*	
C15	−0.2825 (2)	0.13267 (13)	0.44798 (10)	0.0381 (3)	
H15	−0.4314	0.0659	0.4269	0.046*	

### Atomic displacement parameters (Å^2^)

**Table:**

	*U*^11^	*U*^22^	*U*^33^	*U*^12^	*U*^13^	*U*^23^
O	0.0609 (6)	0.0388 (5)	0.0460 (5)	0.0100 (4)	0.0090 (4)	0.0048 (4)
N1	0.0503 (6)	0.0426 (6)	0.0405 (6)	0.0057 (5)	0.0074 (5)	−0.0029 (5)
N2	0.0504 (6)	0.0356 (6)	0.0421 (6)	0.0119 (5)	0.0060 (5)	0.0023 (4)
N3	0.0464 (6)	0.0395 (6)	0.0409 (6)	0.0237 (5)	0.0101 (4)	0.0023 (4)
N4	0.0477 (6)	0.0387 (6)	0.0426 (6)	0.0241 (5)	0.0091 (5)	0.0027 (4)
N5	0.0361 (5)	0.0305 (5)	0.0329 (5)	0.0136 (4)	0.0029 (4)	−0.0026 (4)
C1	0.0359 (6)	0.0367 (6)	0.0293 (5)	0.0039 (5)	0.0025 (4)	−0.0075 (5)
C2	0.0347 (6)	0.0319 (6)	0.0328 (6)	0.0089 (5)	−0.0008 (4)	−0.0063 (4)
C3	0.0314 (5)	0.0339 (6)	0.0333 (6)	0.0118 (5)	0.0034 (4)	−0.0050 (4)
C4	0.0289 (5)	0.0309 (5)	0.0288 (5)	0.0089 (4)	−0.0004 (4)	−0.0076 (4)
C5	0.0348 (6)	0.0356 (6)	0.0336 (6)	0.0149 (5)	0.0018 (4)	−0.0085 (5)
C6	0.0343 (6)	0.0433 (7)	0.0345 (6)	0.0115 (5)	0.0061 (5)	−0.0089 (5)
C7	0.0317 (5)	0.0291 (5)	0.0308 (5)	0.0125 (4)	−0.0002 (4)	−0.0064 (4)
C8	0.0344 (6)	0.0332 (6)	0.0344 (6)	0.0149 (5)	0.0028 (4)	−0.0002 (4)
C9	0.0383 (6)	0.0302 (6)	0.0370 (6)	0.0069 (5)	0.0060 (5)	0.0000 (5)
C10	0.0337 (6)	0.0285 (5)	0.0294 (5)	0.0085 (4)	0.0037 (4)	0.0040 (4)
C11	0.0336 (6)	0.0388 (6)	0.0413 (6)	0.0049 (5)	0.0090 (5)	−0.0032 (5)
C12	0.0430 (7)	0.0369 (7)	0.0440 (7)	−0.0006 (5)	0.0055 (5)	−0.0050 (5)
C13	0.0538 (8)	0.0392 (7)	0.0333 (6)	0.0160 (6)	0.0042 (5)	−0.0033 (5)
C14	0.0415 (7)	0.0581 (8)	0.0365 (6)	0.0159 (6)	0.0111 (5)	−0.0012 (6)
C15	0.0336 (6)	0.0431 (7)	0.0353 (6)	0.0036 (5)	0.0065 (5)	0.0012 (5)

### Geometric parameters (Å, º)

**Table:**

O—N1	1.3804 (15)	C6—H6	0.9500
O—N2	1.3833 (14)	C7—C8	1.3706 (16)
N1—C1	1.3133 (16)	C8—H8	0.9500
N2—C2	1.3173 (15)	C9—C10	1.5107 (15)
N3—N4	1.3112 (14)	C9—H9A	0.9900
N3—C7	1.3633 (14)	C9—H9B	0.9900
N4—N5	1.3452 (13)	C10—C15	1.3821 (16)
N5—C8	1.3370 (14)	C10—C11	1.3833 (16)
N5—C9	1.4582 (15)	C11—C12	1.3845 (17)
C1—C6	1.4237 (17)	C11—H11	0.9500
C1—C2	1.4260 (16)	C12—C13	1.3756 (18)
C2—C3	1.4168 (16)	C12—H12	0.9500
C3—C4	1.3642 (16)	C13—C14	1.3766 (19)
C3—H3	0.9500	C13—H13	0.9500
C4—C5	1.4486 (15)	C14—C15	1.3844 (18)
C4—C7	1.4609 (16)	C14—H14	0.9500
C5—C6	1.3485 (17)	C15—H15	0.9500
C5—H5	0.9500		
			
N1—O—N2	112.34 (9)	C8—C7—C4	130.21 (10)
C1—N1—O	104.58 (10)	N5—C8—C7	105.21 (10)
C2—N2—O	104.48 (10)	N5—C8—H8	127.4
N4—N3—C7	108.93 (9)	C7—C8—H8	127.4
N3—N4—N5	107.19 (9)	N5—C9—C10	112.41 (9)
C8—N5—N4	110.86 (10)	N5—C9—H9A	109.1
C8—N5—C9	128.06 (10)	C10—C9—H9A	109.1
N4—N5—C9	120.90 (9)	N5—C9—H9B	109.1
N1—C1—C6	130.24 (11)	C10—C9—H9B	109.1
N1—C1—C2	109.44 (11)	H9A—C9—H9B	107.9
C6—C1—C2	120.32 (11)	C15—C10—C11	118.90 (11)
N2—C2—C3	129.42 (11)	C15—C10—C9	118.67 (10)
N2—C2—C1	109.17 (11)	C11—C10—C9	122.42 (10)
C3—C2—C1	121.40 (10)	C10—C11—C12	120.27 (11)
C4—C3—C2	117.34 (10)	C10—C11—H11	119.9
C4—C3—H3	121.3	C12—C11—H11	119.9
C2—C3—H3	121.3	C13—C12—C11	120.54 (12)
C3—C4—C5	120.84 (11)	C13—C12—H12	119.7
C3—C4—C7	120.67 (10)	C11—C12—H12	119.7
C5—C4—C7	118.48 (10)	C12—C13—C14	119.45 (11)
C6—C5—C4	123.01 (11)	C12—C13—H13	120.3
C6—C5—H5	118.5	C14—C13—H13	120.3
C4—C5—H5	118.5	C13—C14—C15	120.20 (12)
C5—C6—C1	117.08 (10)	C13—C14—H14	119.9
C5—C6—H6	121.5	C15—C14—H14	119.9
C1—C6—H6	121.5	C10—C15—C14	120.61 (11)
N3—C7—C8	107.80 (10)	C10—C15—H15	119.7
N3—C7—C4	121.94 (10)	C14—C15—H15	119.7
			
N2—O—N1—C1	0.09 (13)	N4—N3—C7—C8	−0.26 (13)
N1—O—N2—C2	−0.13 (13)	N4—N3—C7—C4	177.60 (10)
C7—N3—N4—N5	0.44 (13)	C3—C4—C7—N3	−168.10 (11)
N3—N4—N5—C8	−0.47 (13)	C5—C4—C7—N3	10.64 (16)
N3—N4—N5—C9	−176.07 (10)	C3—C4—C7—C8	9.23 (18)
O—N1—C1—C6	−179.98 (11)	C5—C4—C7—C8	−172.03 (11)
O—N1—C1—C2	−0.02 (12)	N4—N5—C8—C7	0.31 (13)
O—N2—C2—C3	178.79 (11)	C9—N5—C8—C7	175.51 (10)
O—N2—C2—C1	0.11 (12)	N3—C7—C8—N5	−0.03 (12)
N1—C1—C2—N2	−0.06 (13)	C4—C7—C8—N5	−177.65 (11)
C6—C1—C2—N2	179.90 (10)	C8—N5—C9—C10	−61.08 (15)
N1—C1—C2—C3	−178.86 (10)	N4—N5—C9—C10	113.69 (11)
C6—C1—C2—C3	1.10 (16)	N5—C9—C10—C15	149.78 (10)
N2—C2—C3—C4	−178.88 (11)	N5—C9—C10—C11	−31.20 (15)
C1—C2—C3—C4	−0.34 (16)	C15—C10—C11—C12	0.90 (18)
C2—C3—C4—C5	−0.45 (16)	C9—C10—C11—C12	−178.12 (11)
C2—C3—C4—C7	178.26 (9)	C10—C11—C12—C13	0.7 (2)
C3—C4—C5—C6	0.53 (17)	C11—C12—C13—C14	−1.0 (2)
C7—C4—C5—C6	−178.21 (10)	C12—C13—C14—C15	−0.1 (2)
C4—C5—C6—C1	0.22 (16)	C11—C10—C15—C14	−2.06 (18)
N1—C1—C6—C5	178.95 (12)	C9—C10—C15—C14	177.00 (11)
C2—C1—C6—C5	−1.00 (16)	C13—C14—C15—C10	1.69 (19)

### Hydrogen-bond geometry (Å, º)

**Table:**

*D*—H···*A*	*D*—H	H···*A*	*D*···*A*	*D*—H···*A*
C5—H5···N3^i^	0.95	2.50	3.3483 (15)	148
C8—H8···N2^ii^	0.95	2.57	3.4367 (15)	151

Symmetry codes: (i) −*x*+1, −*y*, −*z*; (ii) −*x*, −*y*+1, −*z*.
